# An Immunochemical Approach to Quantify and Assess the Potential Value of the Pseudomonas Quinolone Signal as a Biomarker of Infection

**DOI:** 10.1021/acs.analchem.0c04731

**Published:** 2021-03-11

**Authors:** Enrique J. Montagut, M. Teresa Martin-Gomez, M. Pilar Marco

**Affiliations:** † Nanobiotechnology for Diagnostics (Nb4D), Department of Surfactants and Nanobiotechnology, Institute for Advanced Chemistry of Catalonia (IQAC) of the Spanish Council for Scientific Research (CSIC), 08034 Barcelona, Spain; ‡ CIBER de Bioingeniería, Biomateriales y Nanomedicina (CIBER-BBN), Jordi Girona 18-26, 08034 Barcelona, Spain; § Microbiology Department, Vall d’Hebron University Hospital (VHUH), 08035 Barcelona, Spain; ∥ Genetics and Microbiology Department, Universitat Autònoma de Barcelona (UAB), 08193 Barcelona, Spain

## Abstract

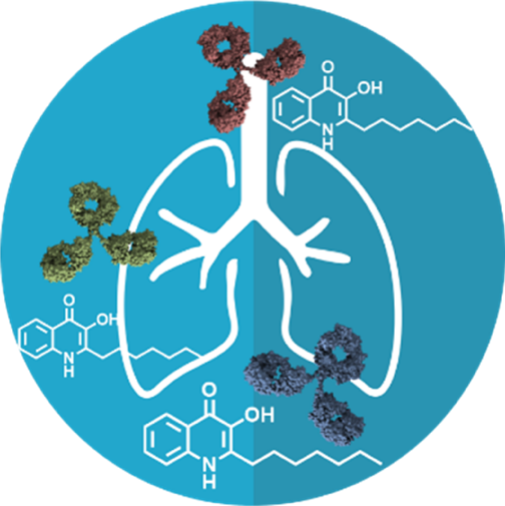

Quorum sensing (QS) is a bacterial cell density-based communication system using low molecular weight signals called autoinducers (AIs). Identification and quantification of these molecules could provide valuable information related to the stage of colonization or infection as well as the stage of the disease. With this scenario, we report here for the first time the development of antibodies against the PQS (pseudomonas quinolone signal), the main signaling molecule from the *pqs* QS system of *Pseudomonas aeruginosa*, and the development of a microplate-based enzyme-linked immunosorbent assay (ELISA) able of quantifying this molecule in complex biological media in the low nanometer range (LOD, 0.36 ± 0.14 nM in culture broth media). Moreover, the PQS ELISA here reported has been found to be robust and reliable, providing accurate results in culture media. The technique allowed us to follow up the PQS profile of the release of bacterial clinical isolates obtained from patients of different disease status. A clear correlation was found between the PQS immunoreactivity equivalents and the chronic or acute infection conditions, which supports the reported differences on virulence and behavior of these bacterial strains due to their adaptation capability to the host environment. The results obtained point to the potential of the PQS as a biomarker of infection and to the value of the antibodies and the technology developed for improving diagnosis and management of *P. aeruginosa* infections based on the precise identification of the pathogen, appropriate stratification of the patients according to their disease status, and knowledge of the disease progression.

Healthcare-associated and community-acquired infections (HAI and CAI, respectively) have become a major burden for public health worldwide,^1^ and *Pseudomonas aeruginosa* is particularly found among the most commonly isolated microorganisms in clinical settings. According to the U.S. Centers for Disease Control and Prevention (CDC), about 51,000 *P. aeruginosa* HAI cases occur each year in U.S. hospitals. This pathogen has developed a vast adaptive response and significant resistance to innate immune effectors and to antibiotics, particularly during chronic infections. Such is the case in patients suffering from cystic fibrosis (CF), a disease caused by a genetic disorder that predominantly affects the mucociliary clearance of the respiratory tract,^2^ creating an ideal scenario for opportunistic pathogens such as *P. aeruginosa*. This pathogen colonizes the lungs of these patients at the age of 2–3 years old and ends up persisting in 60–80% of CF patients as a chronic infection, compromising their life and frequently ending in respiratory failure and premature death.^3^ On the other hand, antibiotic therapy rarely leads to elimination of a pathogen from chronically infected CF respiratory airways. In 2017, multidrug-resistant *P. aeruginosa* caused an estimated 32,600 infections among hospitalized patients and 2700 estimated deaths in the United States.^4^

Recently, the World Health Organization (WHO) has published a list declaring the urgent need of finding new antibiotics for the treatment of infections caused by resistant bacteria, such as carbapenem-resistant *P. aeruginosa*.^5^ The lack of reliable tools for rapid diagnostic of bacterial infections has contributed to the overprescription and misuse of broad spectrum antibiotics, strengthening the generation of multidrug-resistant bacteria.^6^ Current diagnostic methods based on a culture plate require several days to obtain conclusive results, which often is an unaffordable time for effectively treating the infection.^7^ Many diagnostic alternatives have emerged as potential complements of the standard techniques, providing more complete and accurate results. For instance, matrix-assisted laser desorption ionization–time of flight–mass spectrometry (MALDI-TOF-MS) analysis has been successfully used to characterize the protein footprint from bacterial isolates of CF.^8^ Also, polymerase chain reaction (PCR) methods have appeared as interesting alternatives to identify pathogens.^9^ However, these techniques require complex selection of the targets, tedious and time-consuming extractions, highly trained personnel, and/or expensive equipment,^10^ which prevent their wide implementation on clinical settings. Point of care (PoC) devices may be a promising option in future routine clinical diagnostics, providing rapid reliable responses and being low cost and user-friendly to use. PoC performance would allow us to change the management of the patient and would provide faster diagnosis than conventional methods, ideally in almost every place and situation. Most of the time, these devices rely on the use of a biorecognition element that specifically binds the corresponding biomarker target,^11^ such is the case of antibodies.

Host–pathogen interaction involves the release of a large amount of molecules, which have potential as biomarkers for diagnosis, patient stratification, disease monitoring, or for guiding antibiotic treatment as being the subject of investigation.^12,13^ Because of their key role in the development of pathogenesis, the signaling molecules of the quorum sensing (QS) system could be a potential biomarker of infection. QS is a bacterial communication mechanism based on the release of low molecular weight signals that regulate the coordinated genetic expression of a wide array of physiological activities.^14^ These signals, also called autoinducers (AIs), promote their own biosynthesis and their concentration increase as a function of population density. At a particular AI concentration threshold, the genetic expression of decisive factors such as the secretion of virulence factors and biofilm formation is triggered. *P. aeruginosa* QS is a well-studied communication network composed of four systems (*las*, *rhl*, *pqs*, and *iqs*) performing in a complex hierarchical manner. Particularly, the quinolone-based *pqs* system, responsible for the regulation of virulence factors including pyocyanin, elastase, lectin, and rhamnolipids,^15^ has particularly attracted the attention of the scientific community due to its QS-dependent and independent functions.^16^ Its main signaling molecule, 2-heptyl-3-hydroxy-4(1*H*)-quinolone or the PQS (pseudomonas quinolone signal), specific for *P. aeruginosa*, is responsible for important functions besides its signaling activity. For instance, the PQS mediates cytotoxicity and has been indicated to balance the growth and death in *P. aeruginosa* populations.^17^ The PQS also mediates iron acquisition due to its strong chelating character and its activity to prompt the expression of genes related to the biosynthesis of the siderophores pyoverdine and pyochelin.^18,19^ Furthermore, the PQS promotes the formation of outer membrane vesicles (OMVs) due to its remarked lipophilic character. The molecule induces the curvature of the bacterial membrane and resides as an integral component, maintaining its biological activity and achieving the communication between bacteria through the aqueous media.^20^ Eventually, the PQS was also found to modulate the host immune response by dysregulating several defense mechanisms and cytokine expression.^21^

The PQS has been found in several body fluids of infected patients,^22,23^ such as, for example, in sputum, approximately at the micrometer range by thin-layer chromatography (TLC) analysis,^24^ although other more selective techniques found it at concentrations in the low nanometer range.^25^ While mass spectrometry has been the most commonly used technology to quantify these molecules,^26,27^ electrochemical^28^ or bioreporter methods^29^ have also been reported. These measurements require complex sample treatment methods such as extraction using organic solvents and tedious purification or enrichment steps to accomplish the necessary detectability. On the other hand, only in very few cases, these alkylquinolones have been measured in clinical samples, partially due to the increased sample complexity and low detectability attained by some of these techniques. With this scenario, we have focus our attention on the development of specific antibodies against the main signaling molecule of the *pqs* QS system, with the final aim of developing immunochemical diagnostic technologies suitable to assess the potential value of these specific QS molecules as biomarkers of infection. In this paper, we report, for the first time, the development of a highly sensitive microtiter-based enzyme-linked immunosorbent assay (ELISA) to quantify the PQS and its use to analyze the concentration levels of this molecule in culture media where clinical isolates obtained from infected patients have been grown. Preliminary data point to the potential value of this QS molecule as the biomarker to diagnose *P. aeruginosa* infection and to stratify patients in respect to the stage of infection.

## Experimental Section

### General Methods and Instruments

See the Supporting Information.

### Synthesis of PQS Hapten for Immunization

The hapten 3-(2-heptyl-3-hydroxy-4-oxo-1,4-dihydroquinolin-6-yl)propanoic acid **(13)** was synthesized following a similar approach to that described by Reen et al.^30^ for the synthesis of the PQS through a five-step synthetic pathway from 3-(4-aminophenyl)propanoic acid and methyl 3-oxodecanoate **(3)**, obtained by a nucleophilic reaction of 2,2-dimethyl-1,3-dioxane-4,6-dione (Meldrum’s acid) with octanoyl chloride and subsequent methanolysis. All the intermediates and the final product were purified and characterized by nuclear magnetic resonance (NMR) spectroscopy and ultraperformance liquid chromatography (UPLC)-MS/MS.^31^

### Synthesis of the Bioconjugates PQS-KLH and PQS-BSA

A solution of the PQS hapten **(13)** (3.32 mg, 10 μmol) in anhydrous DMF (400 μL) was cooled to 4 °C. Afterward, tri-*n*-butylamine (2.62 μL,11 μmol) and isobutyl chloroformiate (1.56 μL, 12 μmol) were added to the hapten solution and the mixture was left stirring for 15 min at 4 °C and 30 min at room temperature. Then, 200 μL of the reaction mixture was slowly added over the protein solution (bovine serum albumin (BSA) or keyhole limpet hemocyanin (KLH), 2.5 mg/mL, 2 mL in 10 mM phosphate buffered saline (PBS)). Then, the mixture was left stirring for 2 h at RT and overnight at 4 °C without agitation. The bioconjugates were purified by dialysis against 0.5 mM PBS (5 × 5 L) and Milli-Q water (1 × 5 L) and stored freeze-dried at −80 °C. A small fraction (20 μL) of PQS-BSA was kept for MALDI-TOF-MS analysis, rendering a hapten density of 17 haptens per molecule of BSA (see Table S1). Hapten densities of PQS-BSA and PQS-KLH were also assessed by fluorescence, measuring the intensity of the emission at 445 nm, of the native protein and the bioconjugates, and interpolating the value on a calibration curve build with the PQS hapten, rendering the same value of previous MALDI-TOF-MS analysis (see Figure S1).

### Polyclonal Antisera (PAb)

See the Supporting Information.

### ELISA

#### As385/HHQ-BSA ELISA

Microtiter plates were coated with the HHQ-BSA conjugate in coating buffer (0.04 μg/mL, 100 μL/well) overnight at 4 °C and covered with an adhesive plate sealer. The day after, plates were washed with PBST (4 × 300 μL/well) and solutions of PQS standards (2 μM to 0.13 nM in PBST-EDTA, 50 μL/well) followed by the As385 (dil 1/48000 in PBST-EDTA, 50 μL/well) addition and the microplates were left without agitation for 30 min at room temperature. After another washing step, a solution of diluted goat anti-rabbit IgG-HRP (1/6000 in PBST) was added (100 μL/well) and incubated for 30 min at room temperature. The plates were washed again, and the substrate solution (100 μL) was added and left for 30 min at room temperature in the dark. The enzymatic reaction was stopped by adding 4 N H_2_SO_4_ solution (50 μL/well), and the absorbance was read at 450 nm.

#### Immunoassay Evaluation

Performance of the assays was evaluated through the modification of different physicochemical parameters (pH, ionic strength, the presence of a surfactant (% Tween 20), solubility with addition of organic solvents, competence time, incubation time, or cation complexation by EDTA) in the competitive step.

#### Cross-Reactivity Studies

HHQ, PQS, and HQNO calibration curves (10 μM to 1 pM) were built in PBST and measured on the As385/HHQ-BSA ELISA following the procedure described above. The standard curves obtained were fitted to the four-parameter equation mentioned above, and the IC_50_ value was used to calculate the cross-reactivity according to the following equation CR (%) = IC_50_(cross reactant)/IC_50_(analyte) × 100.

### Analysis of Clinical Isolates Culture Samples

#### Samples

Clinical isolates were obtained from lower respiratory tract samples, mainly sputum specimens, of patients diagnosed of acute or chronic respiratory infections. Clinical samples were cultured in MacConkey agar, blood agar, and chocolate agar and incubated for up to 5 days at 37 °C. *P. aeruginosa* isolates were selected and stored frozen at −20 °C. Prior to the analyses, *P. aeruginosa* isolates were cultured overnight at 37 °C in blood agar plates. The day after, a portion of the grown bacteria was diluted in Mueller–Hinton (MH) culture broth (3 mL) and shaken at 37 °C. When the optical density at 600 nm (OD_600_) reached a value of 0.2–0.3, a dilution (1/1000 in 20 mL of MH) was performed and the resulting solution was shaken at 37 °C. Aliquots were taken at the selected times for measurement of OD_600_, for colony forming unit (CFU) calculation, and measurement of the PQS by the ELISA. The rest of the isolates were grown using the same experimental procedure, yet the aliquots were extracted just at a selected time of 8 h. PQS concentrations measured by the ELISA were expressed as PQS immunoreactivity equivalents (IRequiv.) due to the potential specific interferences caused by other alkylquinolones of different chain lengths potentially present in the culture media.

#### Analyses of the Samples Using As385/HHQ-BSA ELISA

Culture broth samples were analyzed following the same procedure as described above except for coating the plates with HHQ-BSA at 0.04 μg/mL (in coating buffer, 100 μL/well) and diluting As385 64,000 times with PBST-EDTA. The standards used to build the standard curves were prepared in MH broth diluted 10 times with PBST-EDTA, and the samples were diluted 10 times with PBST-EDTA before measuring them with the ELISA. To assess the accuracy of the method, blind spiked samples using diluted MH culture broth (1/10) were prepared and measured using the above-described ELISA for three different days and three replicates within the same day. The results are expressed as the mean of all replicates.

## Results and Discussion

Understanding quorum sensing (QS) is key to apprehend about pathogenesis. QS depends on specific signaling molecules or autoinducers, which are responsible for triggering the expression of a series of genes, biosynthesizing virulence factors, and favoring biofilm progression. Rapid detection of these signaling molecules could give clinicians an early indication of infection or knowledge about the stage of the disease.

Over the last few years, biosensors and PoC devices have emerged as promising alternatives for a more rapid and efficient detection of biomarkers of the disease. Among them, antibody-based technologies have demonstrated their capability of providing accurate and reliable diagnostic results thanks to their great affinity, which allows us to develop highly sensitive technologies and their specificity. However, accomplishing high quality antibodies with the necessary affinity and specificity is a crucial factor, which is mainly dependent on the immunogen and of the strategy used to produce such antibodies.

### Synthesis of the Immunizing Hapten

**Figure 1:**
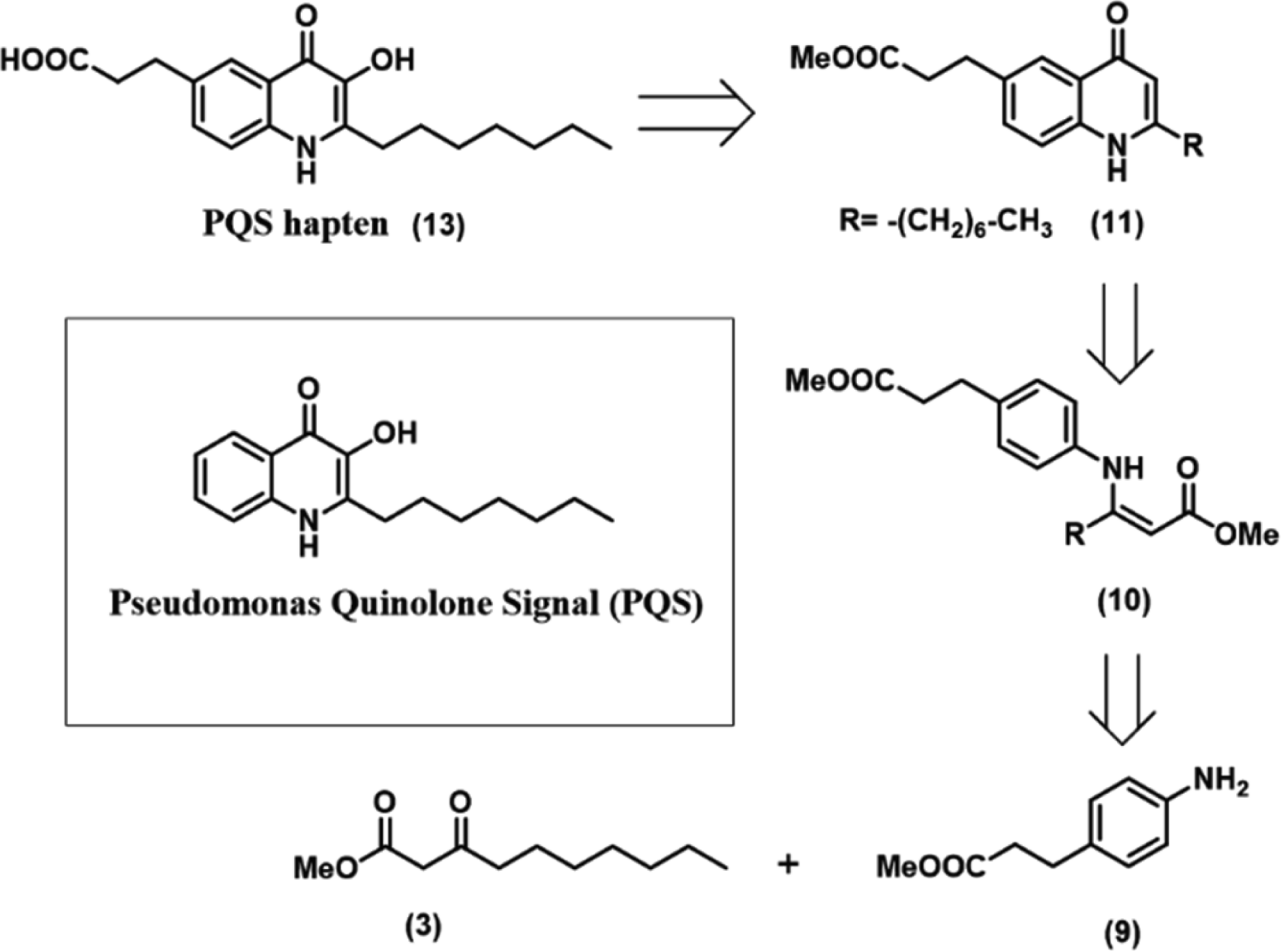
Retrosynthetic scheme for the synthesis of the PQS hapten (8), analogous to the 2-heptyl-3-hydroxy-4(1*H*)-quinolone (PQS) quorum sensing molecule of the *pqs* system from *P. aeruginosa*. The hapten was synthesized through a five-step synthetic pathway from 3-(4-aminophenyl)propanoic acid and methyl 3-oxodecanoate.

The pseudomonas quinolone signal (PQS) is an autoinducer used specifically by *P. aeruginosa* to regulate virulence (see Figure 1) and for this reason constitutes an excellent target to develop new diagnostic and therapeutic strategies. Due to its small size, it is not immunogenic by itself, for which a reason-appropriate immunizing hapten design was performed to ensure the production of high affinity antibodies that would establish strong non-covalent interactions, such as electrostaticity, hydrogen bonds, hydrophobicity, and van der Waals forces, with our target.^32,33^ The quinolone ring and the characteristic catechol moiety of the native PQS structure were considered the most important epitopes, with the capability of establishing such kinds of noncovalent interactions. Therefore, an immunizing hapten was designed incorporating a spacer arm at the C-6 position to promote the exposure of the moiety containing these functional groups. The end of the spacer arm was provided with a carboxylic acid, which eventually allowed the conjugation of the hapten to the lysine residues of an immunogenic protein through the use of orthogonal chemistry (active ester, anhydride mixed, etc.).^34^

The synthesis of the PQS hapten was inspired from a strategy previously described by Pesci and co-workers^35^ based on two sequential modifications of the C-3 position in the PQS biological precursor, 2-heptyl-4-quinolone or HHQ. Hapten synthesis (see Figure 1) started with esterification of 3-(4-aminophenyl)propanoic acid to obtain the protected aniline derivative **(9)**. The aniline derivative was subsequently reacted by amination reaction with β-ketoester **(3)** in the presence of a catalytic amount of *p*-toluene sulfonic acid to obtain the intermediate **(10)**. Afterward, the quinolone derivative **(11)** was prepared through Conrad–Umpach thermic cyclization in diphenyl ether at 270 °C. Formylation of the C-3 position through the Duff reaction^36^ allowed us to obtain the quinolone derivative **(12)** as the precursor of the desired hapten. Concomitant hydrolysis of the methyl ester group occurred; however, this fact no longer affected the synthetic procedure. Finally, oxidation of the C-3 position through Dakin reaction allowed us to obtain the PQS hapten **(13)**. This reaction relies on a characteristic hydrogen peroxide nucleophilic addition that promotes the [1,2]-aryl migration and elimination, subsequently obtaining an hydroxyl group directly bonded to the previously formylated position.^37^ Overall, the pure proposed PQS proposed hapten **(13)** was obtained in five synthetic steps with an overall yield of 16%.

The PQS hapten was used to prepare KLH and BSA protein bioconjugates following the anhydride mixed method. This method relies on the carboxylic acid activation using a chloroformate and a sterically hindered base, forming an anhydride that afterward reacts with the lysine residues present in the protein.^38^ Hapten bioconjugates were obtained with a high density ratio (PQS-BSA ≈ 17; PQS-KLH ≈ 116–130), as demonstrated by MALDI-TOF-MS and fluorescence analysis (Table S1 and Figure S1).

### ELISA for PQS Detection

Antisera (As385, As386, and As387) were increased against PQS-KLH in female New Zealand white rabbits. The avidity of obtained As for the BSA conjugates (HHQ-BSA and PQS-BSA) and the competition of the PQS for the binding to the antibodies were assessed by combined experiments, comparing the absorbance of the assay in the presence and absence of the analyte. After a preliminary selection, the most promising combinations were analyzed by two-dimensional titration experiments. The first set of experiments allowed us to select the best As/bioconjugate combinations for competitive assays based on the largest reduction of the signal when the PQS was present in different combinations, while the second one allowed us to select the concentrations of the immunoreagents providing the best ratio/noise signal. As a result, four different As/bioconjugate combinations were evaluated in a competitive format (see Table S2), from which the combination of As385/HHQ-BSA was selected for further studies due to the higher detectability in further experiments.

The performance of the assay under different physicochemical conditions such as the effect of time, pH, concentration of a non-ionic surfactant, ionic strength, or the presence of an organic solvent was evaluated (see Figure S2). A significant variation of the assay features by introducing a preincubation step was not observed, and the optimum competition time was found to be 30 min.

The concentrations of the BSA conjugate and As dilution used in the assay run in PBST-EDTA buffer were 0.04 μg/mL and 1/48,000, respectively. In the case of the assay run in MH diluted 1/10, the concentrations of the BSA conjugate and As dilution were 0.04 μg/mL and 1/64,000, respectively. The parameters and features of the MH 1/10 curve correspond and refer to the values in the diluted sample. The concentrations are expressed in nanometers, and the data shown correspond to the average of three different days using at least 2 well/replicates per concentration.

The assay was able to work at pH values between 4.5 and 9.5, although a slightly better performance was observed at pH 7.5 based on its detectability and maximum absorbance. The addition of an organic solvent such as DMSO did not produce a significant enhancement; although the *A*_max_ increased, at concentrations greater than 2–5%, the detectability decreased. On the other hand, the sensitivity was dramatically affected by Tween 20. A substantial signal enhancement was observed when Tween 20 was not added to the assay buffer (see Figure S2), while the IC_50_ value was significantly lower (better detectability) than under the standard conditions using a buffer with 0.05% of this detergent. However, since it is known that the presence of a small amount of Tween 20 helps in minimizing nonspecific interactions and solubilizing the analyte, it was decided to keep a concentration of just 0.01%. The detectability was slightly better at conductivity values greater than 15 mS/cm; however, the *A*_max_ decreased substantially, and for this reason, it was decided to keep the conductivity at 15 mS/cm for further experiments. Furthermore, it was added to the assay buffer (0.1 mM) of EDTA as a result of the accuracy experiments (explained in the section below).

### ELISA Evaluation and Characterization

**Figure 2:**
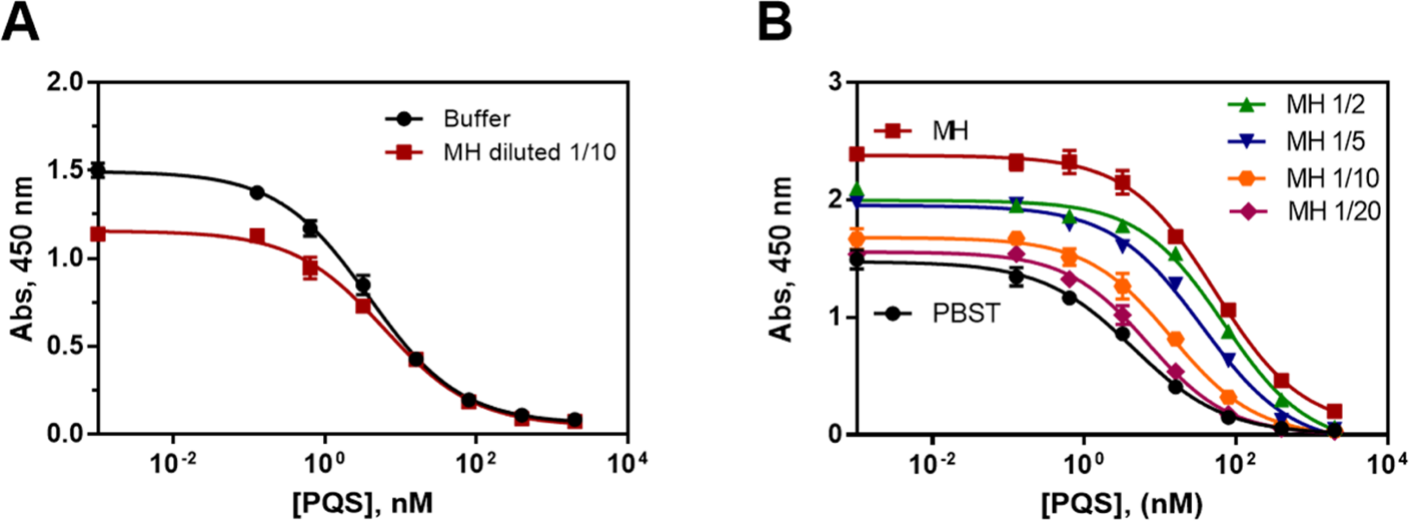
(A) Calibration curves of the As385/HHQ-BSA ELISA for the detection of the PQS run in buffer (PBST-EDTA) and in 1/10 diluted MH broth, under the conditions established (see Table 1). Each calibration point was measured in triplicates on the same ELISA plate, and the results show the average and standard deviation of analysis made on three different days. (B) Matrix effect of MH broth undiluted and diluted 2, 5, 10, and 20 times with PBST on the As385/HHQ-BSA ELISA. The calibration curves were run using the conditions established for the assay in PBST. Modification of the assay conditions allowed us to achieve similar immunoassay features as when the assay was run in buffer (see Table 1 and panel A). The results shown are the average and standard deviations of analysis made on two different days measured by duplicates each day.

Under these conditions, the assay showed an LOD of 0.17 ± 0.01 nM and a dynamic range compressed between 0.53 ± 0.04 and 24.37 ± 3.18 nM (see Table 1 and Figure 2), which is below the average concentrations found in *P. aeruginosa* bacterial cultures (μM) and in the same range of concentrations (low nanometer range) compared to the ones found in sputum samples by Abdalla and co-workers.^25,26,39^ Furthermore, the detectability achieved with the As385/HHQ-BSA ELISA is greater than those obtained with other techniques such as those based on bioreporters^29,40^ or electrochemical methods.^28^ Regarding LC-MS/MS methods, the ELISA reported here allows us to obtain higher or similar detectability without including additional preconcentration steps. These methods require the use of solid phase extraction and/or organic solvent extraction steps, ending up in a 20 times concentration factor of the sample for obtaining the reported LOQs.^22,39^

**Table 1:** Features of the As385/HHQ-BSA ELISA for the Detection of the PQS

	PBST	MH diluted 1/10
*A*_min_	0.03 ± 0.01	0.02 ± 0.01
*A*_max_	1.45 ± 0.04	1.11 ± 0.03
slope	–0.72 ± 0.01	–0.73 ± 0.11
IC_50_	3.87 ± 0.53	6.05 ± 0.16
dynamic range	0.53 ± 0.04 to 24.37 ± 3.18	0.97 ± 0.25 to 36.16 ± 6.20
LOD	0.17 ± 0.01	0.36 ± 0.14
*R*^2^	0.997 ± 0.002	0,995 ± 0,003

The PQS is the most important and active signaling molecule in the *P. aeruginosapqs* QS system. However, other quinolones produced by this pathogen have also an important role in communication or interspecies interaction. The other two molecules are HHQ, the precursor of the PQS in the biosynthetic pathway and involved in the signaling process, and HQNO, an N-oxide quinolone able to inhibit the electron transport chain in other bacteria showing remarkable antistaphylococcal properties.^41^ The only structural difference between HHQ, HQNO, and the PQS is one oxidized position (C-3 or N-1), the reason why the recognition pattern by the As385/HHQ-BSA ELISA had to be assessed. Despite the similarity between the different molecules, the percentage of cross-reactivity was less than 2% for HQNO (IC_50_ = 236.2 nM) and about 13% in the case of HHQ (IC_50_ = 27.2 nM) (see Table S5). Moreover, additional specificity experiments were performed in which calibration curves of the PQS were built under the presence of constant concentrations of either HHQ or HQNO (Figure S3). It allowed us to evaluate the potential cross-reactivity for each point of the calibration curve at different concentrations of cross reactants. The experiments showed that, indeed, the percentages of cross-reactivity were not constant and diminished when the concentrations of the PQS increased. Hence, the ELISA developed recognized a much better PQS despite the great structural similarities of the other two autoinducer molecules of the *pqs* system, which pointed to the possibility to quantify specifically the PQS from samples where all three quinolones will be present. Specificity studies using other molecules structurally related (i.e., quinolone-type antibiotics ciprofloxacin and norfloxacin) and non-structurally related (i.e., the virulence factor pyocyanin or IQS, 2-(2-hydroxylphenyl)-thiazole-4-carbaldehyde, the main signaling molecule of the *iqs* QS system), potentially present in clinical or bacterial culture samples, were also performed. In all cases, the As385/HHQ-BSA ELISA did not respond to concentration values up to 10 μM of all mentioned molecules, and thus, the CR was assumed to be less than 0.01% for all of them.

As385/HHQ-BSA ELISA intra-plate, inter-plate, and inter-day precision were assessed, as it can be observed in Table S3, where the percentage of the coefficient of variation (%CV) at three different concentrations is shown (IC_20_, IC_50_, and IC_80_). The reproducibility is only slightly lower when measuring concentrations close to the limit of quantification during different days, but even at those very low concentration values, the %CV is below near 10% on intra-plate and inter-plate studies.

### ELISA Implementation for Clinical Isolate Analysis

With these results, as a pilot study, the ELISA was used to evaluate the PQS release profile on culture samples where *P. aeruginosa* was grown. The total amount of exo-products and production profile of QS molecules such as the PQS can vary among strains coming from different infection types or infection status^3^ and might have clinical relevance. However, such types of samples may contain a variety of components from the media or from the bacteria, which may interfere with the assay. For this reason, on the first instance, the matrix effect caused by measurement in Mueller–Hinton (MH) culture broth, frequently used to grow *P. aeruginosa* strains, was evaluated. These media contain nitrogenous compounds, vitamins, starch, and amino acids, which added to the products released by the bacteria itself such as virulence factors, proteins, and other exo-products, which could make the sample quite complex. To estimate the extension of this effect, calibration curves were built in MH culture media, at different dilution factors, and measured with the As385/HHQ-BSA ELISA. The resulting calibration curves and the effect caused by MH in the assay performance can be seen in Figure 2B. The measurement in MH produced a substantial increase of the maximum signal and a decrease in the detectability of the assay. This effect could be diminished by increasing the dilutions of the MH media with the assay buffer; however, the same features as the assay run in buffer could not be reached unless the MH broth was diluted at least 20 times, which compromised too much the detectability for our purposes. In light of this behavior, two-dimensional titration experiments were carried out using MH media diluted 10 times with the assay buffer to set up new assay conditions in respect to the concentration of immunoreagents. As it can be seen in Figure 2A, the assay run in MH diluted 1/10 provided very good features in a similar range to the ones obtained in buffer. The IC_50_ value was 6.05 ± 0.16 nM, while the obtained LOD was 0.36 ± 0.14 nM (Table 1), which taking into account, the dilution factor would turn into 60.5 ± 1.6 and 3.6 ± 1.4 nM, respectively. The PQS is normally found in the micrometer range^39^ in *P. aeruginosa* cultures, and thus, the decrease in detectability caused by the MH dilution factor did not have to mean a negative impact on the PQS production kinetics assessment, allowing the quantification from a considerably low concentration.

**Figure 3:**
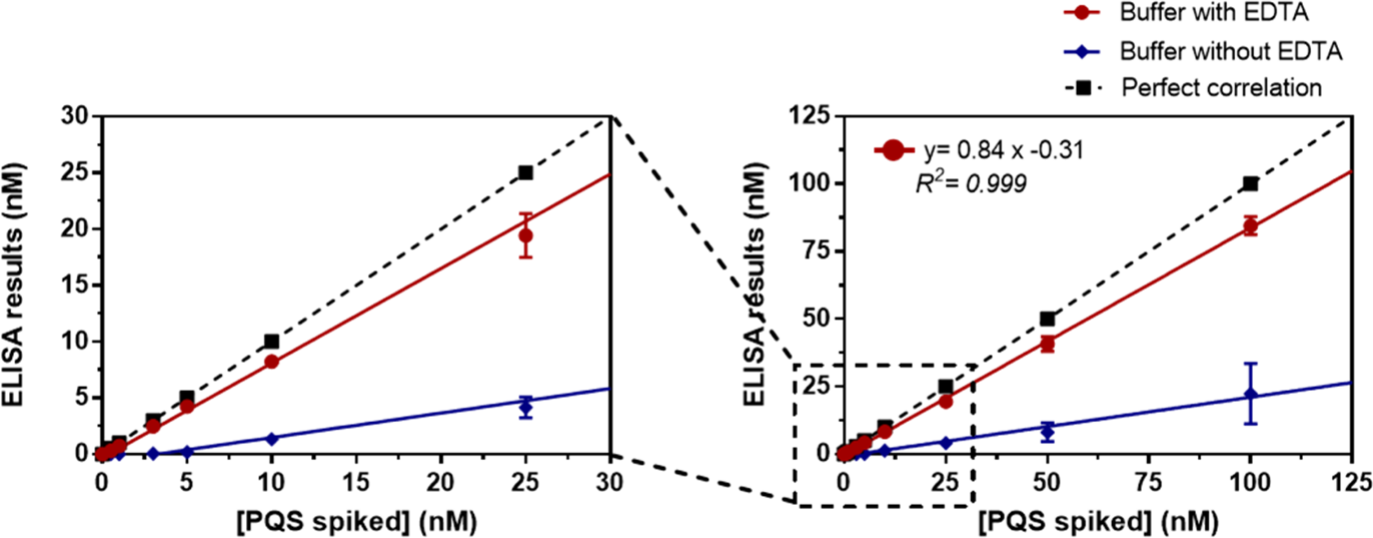
Results from the accuracy study. The graph shows the linear regression analysis of the concentration of the PQS spiked in MH broth and the concentration measured with the As385/HHQ-BSA ELISA. Assays were run in diluted MH culture media 1/10 using PBST (blue line) and PBST-EDTA (red line). Each calibration point was measured in triplicates on the same ELISA plate, and the results show the average and standard deviation of analysis made on three different days.

Accuracy of the assay was evaluated by preparing blind spiked samples in MH culture broth and measuring them with the developed ELISA. Interestingly, the results showed a critical underestimation of the spiked PQS concentrations (Figure 3). We hypothesized that this effect might be caused by the described chelating properties of the PQS. Thus, it is known that the role of PQS chelating ferric iron (Fe^3+^) in a non-deliverable manner^19^ causes an iron starvation response and stimulates a concentration-dependent increase production of iron scavenging siderophores such as pyoverdine and pyochelin. PQS-Fe^3+^ has been found to accumulate in the membrane of the cell, acting as a storage molecule. Considering such behavior, the PQS might be forming complexes with the cations present in MH culture broth. To probe this hypothesis, the calibration curve was run in the presence of different concentrations of ethylenediaminetetraacetic acid (EDTA), a powerful ion chelator able to displace the divalent cation complexation by the PQS (Figure S4). The addition of EDTA caused a substantial increase of the maximum absorbance; nevertheless, it produced an increase in detectability. In light of these results, we assessed the effect of EDTA in the buffer (PBST-EDTA) on the assay accuracy. As it is shown in Figure 3, EDTA produced a significant improvement of the accuracy achieving a much better correlation between spiked concentrations and the value measured with the ELISA. These results indicate that the PQS in the form of a complex is not recognized by the antibodies in the same manner as in the free form. Thus, subsequent studies with the As385/HHQ-BSA ELISA were performed in the presence of EDTA in assay buffer.

With these results, we addressed the investigation of the PQS production profile of clinical isolate growth on MH broth media. Since previously performed experiments pointed at a different production profile of HHQ, the PQS biosynthetic precursor, depending on whether the clinical isolates belonged to patients with a chronic or an acute infection, and the PQS profiles of the release of isolates from patients with an acute (PAAI6) and a chronic (PACI6) *P. aeruginosa* airway infection, respectively, were initially measured.

**Figure 4:**
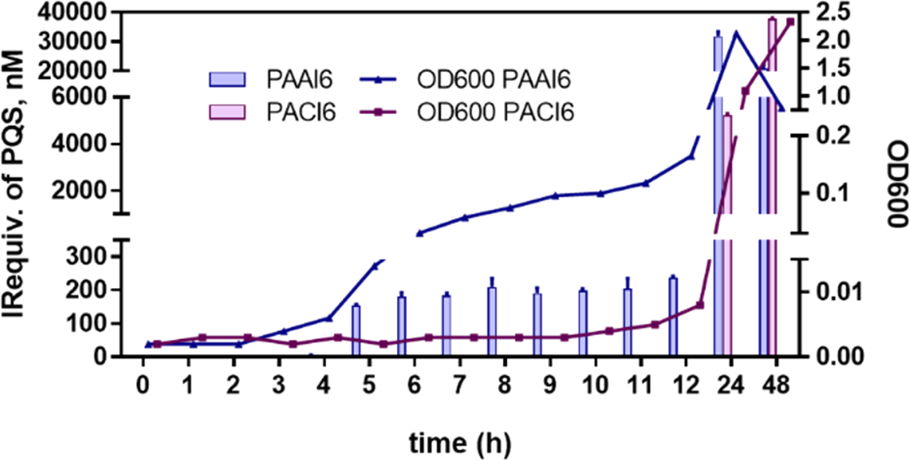
Bacterial growth, expressed as OD_600_ and PQS immunoreactivity equivalents (IRequiv.) measured in MH broth media where *P. aeruginosa* clinical isolates PAAI6 and PACI6 were cultured. Samples were taken at the selected times and measured using the As385/HHQ-BSA ELISA. Each calibration point was measured in triplicates on the same ELISA plate, and the results show the average and standard deviation of analysis made on two different days.

Interestingly, similarly to HHQ, PQS quantifiable levels were reached after 5 and 12 h of growth for the clinical isolates PAAI6 and PACI6, respectively, while after 48 h, the PQS IRequiv. value of PAI6 was higher than that of PAAI6 (see Figure 4), as already observed for HHQ. Moreover, the PQS release correlated to the growth, as evidenced by the OD_600_ and the calculated CFUs. The decrease in the OD_600_ at 48 h of growth observed in PAAI6 might be explained by the prolonged exposure to high concentrations of self exo-products with lytic activity, such as pyocyanin and the PQS.^42−44^

**Figure 5:**
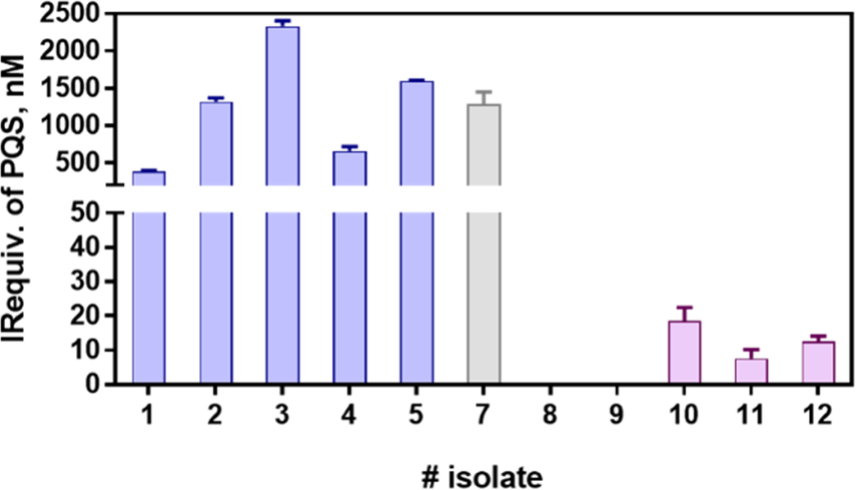
PQS IRequiv. recorded from a collection of clinical isolates from patients with different clinical profiles. Samples were grown in MH broth for 8 h, and the aliquots taken were diluted five times with PBST-EDTA prior to the ELISA analyses. Clinical isolates 1–5 were obtained from patients undergoing acute infection, and isolates 8–12 were obtained from patients undergoing chronic infection. Isolate number 7 corresponds to the reference strain PAO1. The reference number of clinical isolates can be found in Table S7. Each calibration point was measured in triplicates on the same ELISA plate, and the results show the average and standard deviation of analysis made on two different days.

The next set of experiments was seeking to demonstrate that this was a general behavior for clinical isolates coming from patients suffering from *P. aeruginosa* infection with distinct severity degrees. For this purpose, clinical isolates from 12 patients with acute or chronic infections were grown in MH at 37 °C. After 8 h (time chosen as a compromise based on the above-described behavior), sample aliquots were taken and measured with the PQS ELISA developed. As it can be observed in Figure 5, high PQS IRequiv. values (0.5–3 μM) were measured on the culture media from isolates belonging to patients (1–5) with an acute infection, while the PQS IRequiv. values of the isolates from patients (8–12) with a chronic infection were much lower (nanometer range) or even below the LOD under conditions used.

In light of this outcome, it seems clear that the concentration of QS molecules released to the media can be correlated with the type of infection or the disease status of the patient. It has been often reported that the behavior of strains inducing acute or chronic infections is substantially different in terms of virulence and exo-product release caused by an adaptation to the hostile host environment. Thus, on chronic lung infections, *P. aeruginosa* adapts to the host environment by evolving toward a state of reduced bacterial invasiveness that favors bacterial persistence without causing overwhelming host injury. In that respect, the results found with the here reported PQS ELISA are in agreement with this situation. The low levels of the PQS released by the isolates from chronic infected patients would justify a potential lower production of virulence factors. From a diagnostic perspective, in addition to identifying the microorganisms causing the disease, being able to distinguish between a chronic and an acute infection is very relevant to apply appropriate therapeutic strategies and improve disease management.

## Conclusions

In this work, for the first time, antibodies against the most relevant and biologically active quinolone from the *pqs* QS system of *P. aeruginosa* have been reported. The produced antibodies were used to develop the indirect competitive As385/HHQ-BSA ELISA for the quantification of the PQS, achieving a detectability that is within the range of concentrations found in biological and clinical matrices. The results obtained when measuring the PQS IRequiv. released by clinical isolate growth on MH culture broth demonstrated the ability of the As385/HHQ-BSA ELISA to stratify patients depending on the state of the disease. If this immunochemical strategy is applied for a higher number of QS molecules and/or infection biomarkers, then it might provide interesting information for diagnostic or prognostic purposes as well as about the pathogenic strain, the status of the patient, and/or the progression of the disease. In such a case, the technique developed in this work could be envisaged as a future complement of the standard clinical analysis and help clinicians on taking decisions about the treatment and the management of infected patients. On the other hand, the obtained limits of detection and dynamic ranges led us to consider applying the method for the direct quantification of the PQS in clinical respiratory samples from infected patients and its evaluation as the biomarker of infections. Eventually, the versatility and specificity of antibodies would also allow us to implement the developed technique in a wide variety of sensors and/or PoC devices, supporting the rapid and straightforward diagnostic of infections caused by *P. aeruginosa* and subsequently improving the management of the patients.

## Supporting Information Available

Immunogen and coating antigen preparation, competitive assay selection, physicochemical parameter optimization, cross-reactivity studies, and characterization clinical isolates (PDF)
