# Supplementary material for: An Immunochemical Approach to Quantify and Assess the Potential Value of the Pseudomonas Quinolone Signal as a Biomarker of Infection
Source: Anal Chem. 2021 Mar 11;93(11):4859–66. doi: 10.1021/acs.analchem.0c04731 (PMC8479725; doi:10.1021/acs.analchem.0c04731)
Supplement: Supplementary file 1 — ac0c04731_si_001.pdf [file ac0c04731_si_001.pdf]

Supporting Information

An Immunochemical Approach to Quantify and Assess the Potential Value of Pseudomonas Quinolone Signal (PQS) as a Biomarker of Infection

Enrique-J. Montagut[A,B], M. Teresa Martin-Gomez[C,D] and M.-Pilar Marco[A,B]*

A. Nanobiotechnology for diagnostics (Nb4D), Department of of Surfactants and Nanobiotechnology, Institute for Advanced Chemistry of Catalonia (IQAC) of the Spanish Council for Scientific Research (CSIC), Spain.

B. CIBER de Bioingeniería, Biomateriales y Nanomedicina (CIBER-BBN), Jordi Girona 18-26, 08034 Barcelona, Spain

C. Microbiology Department, Vall d'Hebron University Hospital (VHUH), Barcelona, Spain

D. Genetics and Microbiology Department, Universitat Autònoma de Barcelona (UAB), Barcelona, Spain

*To whom correspondence should be sent:

M.-Pilar Marco
Nanobiotechnology for Diagnostics (Nb4D) group
Department of Surfactants and Nanobiotechnology

IQAC-CSIC

Jordi Girona 18-26, 08034 Barcelona, Spain

Spain

Phone: +34 93 4006100

FAX: +34 93 2045904

E-mail: pilar.marco@cid.csic.es

TABLE OF CONTENTS:

General materials and methods S-3

Hapten density analysis of the bioconjugates S-4

Antibody production S-5

Immunochemical assays S-5

Physicochemical parameters optimization S-8

PQS production in clinical isolates S-11

GENERAL MATERIALS AND METHODS

Chemistry: The chemicals used in the synthesis of the haptens were obtained from Sigma Chemical Co. (St. Louis, MO, USA), Aldrich Chemical Co. (Milwaukee, WI, USA), or Acros Organics B.V.B.A. (Morris Plains, NJ, USA). ^{1}H and ^{13}C NMR spectra were obtained with a Varian Mercury-400 spectrometer (400 MHz ^{1}H and 101 MHz for ^{13}C). Thin-layer chromatography (TLC) was performed on 0.25 mm, pre-coated silica gel 60 F254 aluminium sheets (Merck, Darmstadt, Germany). Liquid chromatography/electrospray ionization/mass spectrometry (LC/ESI/MS) was performed in a Waters (Milford, MA, USA) model composed by an Acquity UPLC system directly interfaced to a Micromass LCT Premier XE MS system equipped with an ESI LockSpray source for monitoring positive and negative ions. Data were processed with MassLynx (V 4.1) software (Waters).

Immunochemistry: Chemicals and biochemicals were obtained from Sigma Chemical Co. (St. Louis, MO, USA) and from Aldrich Chemical Co. (Milwaukee, WI, USA). The stock solutions of the alkylquinolones (PQS, HHQ: 2-heptyl-4-quinolone and HQNO: 2-hepty-4-hydroxyquinoline N-oxide) used as standards were prepared in DMSO at 10 mM and stored at -20ºC, then transferred to 4ºC prior to their use. Purification of protein conjugates was carried out by dialysis using Spectra/Pore membranes from Spectrum labs (Piraeus, Greece, EU) with molecular weight cut-off of 12-14 kDa or either using size exclusion HiTrap desalting columns on an ÄKTA Prime Plus Liquid Chromatograpy system (both from GE Healthcare (Chicago, IL, USA). The matrix-assisted laser desorption ionization time-of-flight mass spectrometer (MALDI-TOF-MS) was a Bruker autoflex III Smartbeam spectrometer (Billerica, Massachusetts, US). The pH and the conductivity of all buffers and solutions were measured with a pH-meter pH 540 GLP and a conductimeter LF 340, respectively (WTW, Weilheim, Germany). Polystyrene microtiter plates used for the ELISAs were purchased from Nunc (Maxisorp, Roskilde, Denmark). Dilution plates were purchased from Nirco (Barberà del Vallés, Spain). Washing steps were performed on a Biotek ELx465 (Biotek Inc.). Absorbances were read on a Thermo Scientific MultiSkan GO (Thermo Fisher Scientific, Waltham, MA, USA) at a single wavelength mode (450 nm). The competitive curves were analyzed with a four-parameter logistic equation using the software GraphPad Prism 7.0 (GraphPad Software Inc., San Diego, CA, USA) according to the following formula: $y = B(A-B)/[1 - (x/C)^{D}]$, where A is the maximum absorbance, B is the minimum absorbance, C is the concentration producing 50% of the maximal absorbance, and D is the slope at the inflection point of the sigmoid curve. Unless otherwise indicated, the data presented correspond to the average of at least two well replicates.

HAPTEN DENSITY ANALYSIS OF THE BIOCONJUGATES

Hapten Density Analysis of the PQS-KLH Bioconjugate. The PQS-KLH conjugate hapten density calculation was performed by fluorescence based on the fluorescent properties of PQS and the corresponding hapten. Both compounds showed an emission band at 445 nm when using an excitation wavelength of 340 nm. The conjugation degree was evaluated by interpolating the fluorescence signal intensity of PQS-KLH bioconjugate solutions in a linear regression curve build with the fluorescence signal intensity of distinct PQS hapten standard solutions (see Fig. S2). The number of conjugated residues was calculated by iteration using initially the KLH molecular weight (350-400kDa), rendering a hapten density of 116-130 haptens per molecule of KLH (see table S1).

Hapten Density Analysis of the PQS-BSA Bioconjugate*.* Hapten densities of BSA conjugate were calculated *by* fluorescence as it has been described above and by MALDI-TOF-MS. For the last case, hapten densities were calculated by comparing the molecular weight recorded on the MALDI spectra of the native proteins to that of the PQS-BSA bioconjugates. For this purpose, the bioconjugates were mixed with the freshly prepared matrix ((trans-3,5-dimethoxy-4-hydroxycinnamic acid, 10 mg mL^{-1} in 70:30 ACN/H2O, 0.1% HCOOH) following the ''sandwich'' sample preparation method. The bioconjugate aliquot is diluted ½ using ACN with HCOOH 0.2%. According to it, the matrix (2 µL) is deposited on the MALDI plate and dried, followed by the bioconjugate solution (2 µL, 2 to 5 mg mL^{-1} in 1:1 ACN/H2O, 0.1% HCOOH), allowed to dry again and finally, the matrix solution (2 µL) was added over again. The resulting dried spot was then analyzed by MALDI-TOF-MS. Hapten densities were calculated through the equation: [MW(conjugate) − MW(native protein)]/[MW(hapten)-MW(lost atoms)].

Table S1. Data on the bioconjugation yield and hapten densities of the PQS bioconjugates.

	**Quantity (mg)**	**Yield (%)**	**Hapten density**
**PQS-BSA**	5.95	119	17
**PQS-KLH**	5.29	105	116-130

The hapten density wwas calculated by MALDI-TOF (PQS-BSA) and by fluorescence assays (PQS-BSA and PQS-KLH).

.

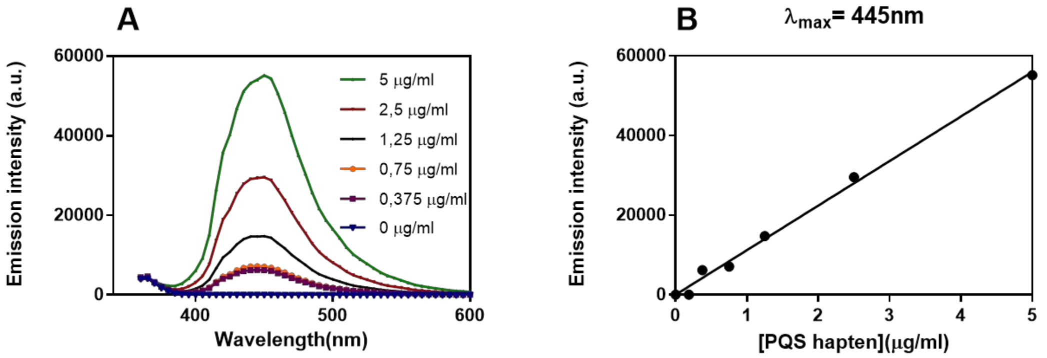

Figure S1. A. Emission spectra at different concentrations of PQS hapten (13) in PBS 10 mM buffer using a λ_{exc} of 340 nm. **B**. Linear regression of the emission intensity at 445 nm versus the concentration of PQS hapten (13) using a λ_{exc} of 340 nm.

ANTIBODY PRODUCTION

Polyclonal antisera (PAs). Antibody production has been performed with the support of the ICTS "NANBIOSIS", more specifically by the Custom Antibody Service (CAbS, CIBER-BBN, IQAC-CSIC)**.** Three female New Zealand white rabbits (385, 386 and 387) weighing 1–2 kg were immunized with PQS-KLH following procedures already reported[1]. Immunizations were carried out in the animal facility of the Research and Development Center (CID) of the Spanish Research Council (CSIC) Registration Number: B9900083, employing approved procedures that avoid unnecessary treatments and minimize suffering of the animals. The protocol used in accordance with the institutional guidelines under a license from the local government (DAAM 7463) and approved by the Institutional Animal Care and Use Committee at the CID-CSIC. The antisera (As) obtained were named As385, As386 and As387. The animals were exsanguinated after 6 immunizations, and the final blood was collected in vacutainer tubes provided with a serum separation gel. Antisera were obtained by centrifugation at 4 °C for 10 min at 10 000 rpm, then stored at −80 °C in the presence of preservative 0,02% sodium azide. The antibody titer was assessed during the immunization process through non-competitive indirect ELISA. Microtiter plates were coated with a fixed concentration of PQS-BSA conjugate (1 mg mL^{-1}) and the avidity of the produced antibodies was measured by preparing serial dilutions of the corresponding As (As385, As386 and As387).

IMMUNOCHEMICAL ASSAYS

Buffers. Unless otherwise indicated, coating buffer is a 50 mM bicarbonate-carbonate buffer (pH 9.6). Phosphate buffer saline (PBS) corresponds to 10mM phosphate buffer and 0.8% saline solution (pH 7.5). PBST is PBS with 0,05% Tween 20 (pH 7.5). PBST-EDTA is PBS with 0.01% Tween 20 and 0.1 mM EDTA. Citrate buffer corresponds to a 40 mM sodium citrate solution (pH 5.5). The substrate solution contains 0.01% of 3,3',5,5'- tetramethylbenzidine (TMB) and 0.004% H_2O_2 prepared in citrate buffer. All buffers were prepared using ultra-pure Milli-Q® water with a resistivity between 16-18 MΩ cm.

Non-competitive indirect two-dimensional titration experiments. Non-competitive indirect ELISA were carried out to select the As dilution and the concentrations of the bioconjugates (PQS-BSA and HHQ-BSA) used as coating antigens (CA). With this regard, the binding of serial dilutions of the antisera (As385-387, 1/1000 to 1/64000, and zero in PBST, 100 µL/well) to microplates coated with the BSA bioconjugates (PQS-BSA and HHQ-BSA[2], 5 µg mL^{-1} to 5 ng mL^{-1}, and zero in coating buffer, 100 µL/well) were measured for all As/bioconjugate combinations, each of them, on different ELISA microplates. From these experiments, suitable concentrations for bioconjugates and antisera dilutions were chosen to produce around 0.8-1.5 units of absorbance after 30 min of competitive step.

Table S2. Analytical parameters of the competitive indirect ELISAs obtained for PQS detectión[a]

**As#, dil➔**	**As 386; 1/8000**	**As 385; 1/16000**	**As 385; 1/32000**	**As 387; 1/16000**
**[CA] (µg/ml)➔**	HHQ-BSA; 0.16	HHQ-BSA; 0.04	PQS-BSA; 0.08	PQS-BSA; 0.31
Bottom	0.12	0.06	0.03	-
Top	1.25	2.03	1.73	-
Hill Slope	-0.72	-0.77	-0.73	-
IC_{50}	24.1	18.5	19.1	-
R^2	0.991	0.999	0.995	-

[a] PQS was used to build standard curves which were measured with the different As/bioconjugate combinations, using the same procedure reported in the main body of the article for the As385/HHQ-BSA ELISA, but with the concentrations of immunoreagents shown in the table, and using PBST in the competition step.

Table S3. Coefficients of Variation (CV) of the As385/HHQ-BSA ELISA run in MH culture broth diluted 1/10 using representative concentrations at a low, medium and high concentration range (IC_{20}, IC_{50} and IC_{80}) .

	IC	R1	R2	R3	μ	σ	%CV
**Inter-day**	**20**	31,45	35,30	40,59	35,78	4,59	12,8
	**50**	6,29	5,27	6,17	5,91	0,56	9,4
	**80**	1,30	0,80	0,93	1,01	0,26	25,4
**Inter-plate**	**20**	38,33	44,91	38,36	40,53	3,79	9,4
	**50**	5,51	5,95	6,04	5,84	0,28	4,8
	**80**	0,79	0,74	0,92	0,82	0,09	11,6
**Intra-plate**	**20**	29,93	30,21	31,68	30,61	0,94	3,1
	**50**	5,88	6,37	6,51	6,25	0,33	5,3
	**80**	1,09	1,20	1,28	1,19	0,09	7,9

The coefficient of variation (CV) was calculated following the equation CV (%) = $\sigma/\mu \times 100$. The results were obtained by measurements performed in either triplicates on the same ELISA plate (intra-plate), made on three different days (inter-day) or by analysis on three different plates (inter-plate). The concentrations of the replicates, mean, standard deviation and ICs are expressed in nM. IC: Inhibitory Concentration; Rn: Replicate; σ: Standard Deviation; μ: Average.

PHYSICOCHEMICAL PARAMETERS OPTIMIZATION:

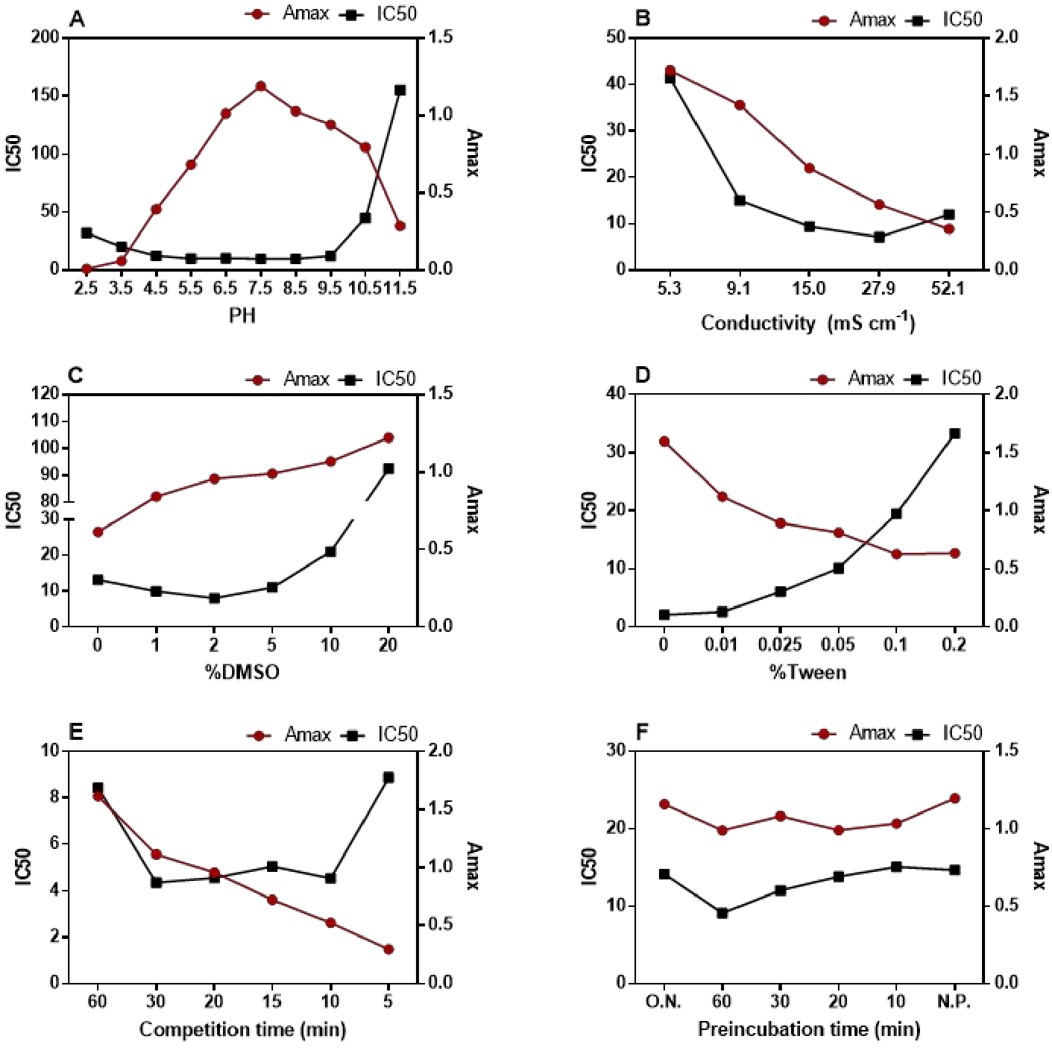

Figure. S2. As385/HHQ-BSA ELISA performance in the physicochemical parameters optimization study. The selection of the most appropriate conditions (Table S2) was based on the variations in Amax, IC50 and slope values (not shown) of the generated calibration curves providing better signal/noise ratio, detectability and sensitivity. The studied parameters were **A.** pH **B.** Ionic Strength **C.** % organic solvent (DMSO) **D.** % Tween20 **E.** competition time **F.** preincubation time. All the studies were performed by varying the composition of the buffer used in the competitive step or the antibody detection times. Eventually, the conditions providing better features were evaluated again separately and in conjunction.

Table S4. Physicochemical parameters selected for the As385/HHQ-BSA ELISA run in buffer.

	As385 HHQ-BSA
**As dilution**	1/32000
**[Competitor] (µg mL^{-1})**	0.04
**pH**	7.5
**Conductivity (mS cm^{-1})**	15
**Tween 20 (%)**	0.01
**Competition time (min)**	30
**Preincubation time (min)**	0
**Organic solvent (%)**	0

The parameters improving the features of the assay were assessed separately and in conjunction.

Table S5. Results of the specificity studies of the As385/HHQ-BSA ELISA

	HHQ	PQS	HQNO	PYO	IQS	Ciprof.	Norf.
**A_{min}**	0.07	0.05	0.08	1.58	1.61	1.51	1.55
**A_{max}**	1.64	1.46	1.63	1.53	1.64	1.55	1.49
**Slope**	-0.66	-0.52	-0.80	-	-	-	-
**IC_{50} (nM)**	27.2	3.6	236.2	-	-	-	-
**R^2**	0.987	0.987	0.993	-	-	-	-
**CR (%)**	13	100	2	<0.01	<0.01	<0.01	<0.01

Standards solutions (2 µM to 0.13 nM and zero in PBST-EDTA) of *P. aeruginosa* QS molecules (PQS, HHQ, HQNO, IQS) and a virulence factor (PYO), as well as structurally related antibiotics (Ciprofloxacin and Norfloxacin) that could potentially co-exist in the media, were used to prepare calibration curves which were measured with the ELISA to assess potential specific interferences caused in the assay. The percentages of cross reactivity (C.R.) were calculated following the equation: CR (%) = IC_{50}(PQS)/IC_{50}(Assessed compound) x 100. PYO: pyocyanin; IQS: (2-(2-hydroxylphenyl)-thiazole-4-carbaldehyde); Ciprof.: Ciprofloxacin; Norf: Norfloxacin.

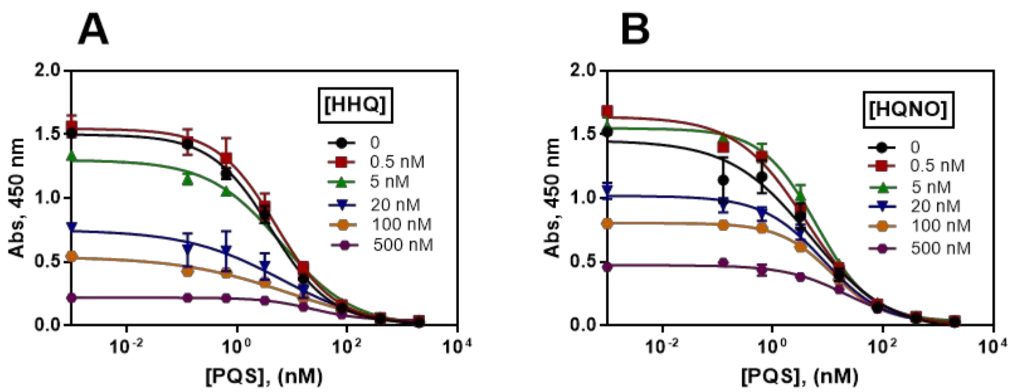

Figure S3. Calibration curves and evaluation of the simultaneous presence of PQS and different cross reactants concentrations in the As385/HHQ-BSA ELISA. **A.** Effect of constant concentrations of HHQ in the different calibration points. **B.** Effect of constant concentrations of PQS in the different calibration points. Calculated cross reactivity was equal or less than the

reported percentages of CR in the previous specificity assays for the calibration points within the dynamic range. Each calibration point was measured in duplicates on the same ELISA plate and the results show the average and standard deviation of analysis made on two different days.

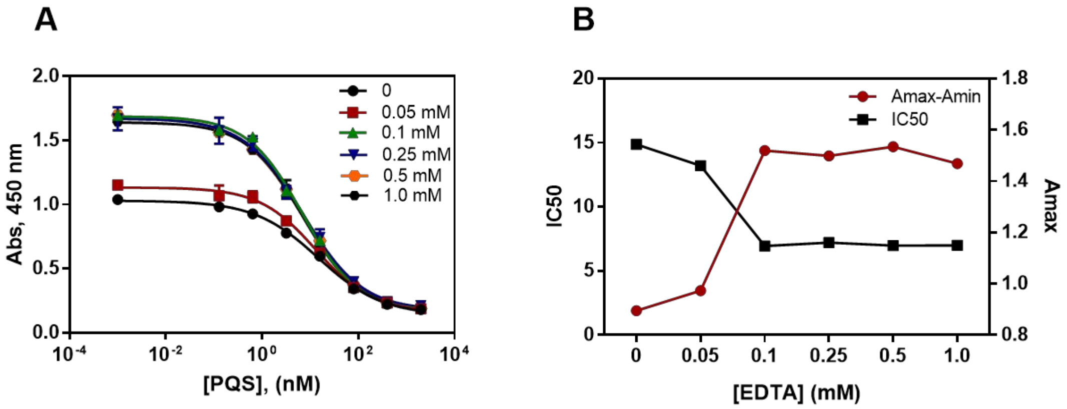

Figure S4. **A**. Calibration curves of As385/ HHQ-BSA ELISA competitive indirect assay run in PBST buffer under the presence of different EDTA concentrations. **B**. Representation of the IC50 and maximum absorbance for the As385/ HHQ-BSA ELISA versus the assessed concentrations of EDTA.

PQS PRODUCTION IN CLINICAL ISOLATES:

Matrix effect studies. MH culture broth media diluted in PBST-EDTA (1:2, 1:5, 1:10, 1:20) was used to prepare PQS standard calibration curves and to compare them with the standard curves prepared in the PBST-EDTA buffer.

Table S6. Colony Forming Units (CFUs) calculated using the formula described by Dong-Ju Kim et al[3]. using the optical density measured at 600 nm (OD_{600}) for clinical isolates PAAI6 and PACI6.

t (h)	OD_{600} acute	CFUs acute	OD_{600} chronic	CFUs chronic
**0**	0.002	4,4E+06	0.002	4,4E+06
**1**	0.003	4,6E+06	0.002	4,4E+06
**2**	0.003	4,6E+06	0.002	4,4E+06
**3**	0.004	4,8E+06	0.002	4,4E+06
**4**	0.006	5,2E+06	0.003	4,6E+06
**5**	0.014	6,8E+06	0.002	4,4E+06
**6**	0.031	1,0E+07	0.003	4,6E+06
**7**	0.058	1,6E+07	0.003	4,6E+06
**8**	0.075	1,9E+07	0.003	4,6E+06
**9**	0.096	2,3E+07	0.003	4,6E+06
**10**	0.100	2,4E+07	0.004	4,8E+06
**11**	0.118	2,8E+07	0.005	5,0E+06
**12**	0.165	3,7E+07	0.008	5,6E+06
**24**	2.125	-	1.096	2,2E+08
**48**	0.796	1,6E+08	2.333	-

Table S7. Clinical isolates reference number and concentration of PQS measured with the developed As385/ HHQ-BSA ELISA.

#Number	#Ref	Infection type	[PQS](nM)	Desv. Est. (nM)
**1**	**PAAI1**	Acute	387.6	10.37
**2**	**PAAI2**	Acute	1320.4	51.14
**3**	**PAAI3**	Acute	2333.3	75.32
**4**	**PAAI4**	Acute	658.85	62.32
**5**	**PAAI5**	Acute	1599.8	5.91
**6**	**PAAI6**	Acute	210.0	25.16
**7**	**PAO**	Reference	1286.5	161.74
**8**	**PACI1**	Chronic	LLOQ	-
**9**	**PACI2**	Chronic	LLOQ	-
**10**	**PACI3**	Chronic	18.70	3.81
**11**	**PACI4**	Chronic	7.63	2.59
**12**	**PACI5**	Chronic	12.63	1.58
**13**	**PACI6**	Chronic	LLOQ	-

The clinical isolates were grown 8 hours in MH culture media at 37ºC following the protocol described in the main article. Sample aliquots were taken and after a dilution of 5 times with PBST-EDTA, the samples were analyzed by ELISA using the procedure described in the experimental section of the main body of the article. Clinical isolates 1-5 were obtained from patients undergoing acute infection and isolates 8-12 were obtained from patients undergoing chronic infection. Isolate number 7 corresponds to the reference strain PAO1. The reference number of clinical

isolates can be found in Table S7. Each calibration point was measured in triplicates on the same ELISA plate and the results show the average and standard deviation of analysis made on two different days.

SUPPORTING INFORMATION REFERENCES

1. Marco, M. P.; Nasiri, M.; Kurth, M. J.; Hammock, B. D., Enzyme-linked immunosorbent assay for the specific detection of the mercapturic acid metabolites of naphthalene. *Chemical Research in Toxicology* **1993,** *6* (3), 284-293.
2. Montagut, E. J.; Vilaplana, L.; Martin-Gomez, M. T.; Marco, M. P., High-Throughput Immunochemical Method to Assess the 2-Heptyl-4-quinolone Quorum Sensing Molecule as a Potential Biomarker of Pseudomonas aeruginosa Infections. *ACS Infect Dis* **2020,** *6* (12), 3237-3246.
3. Kim, D.-j.; Chung, S.-g.; Lee, S.-h.; Choi2, J.-w., Relation of microbial biomass to counting units for Pseudomonas aeruginosa. *African J. Microbiol. Res.* **2012,** *6* (21), 4620-4622.
